# Understanding and overcoming resistance to immunotherapy in genitourinary cancers

**DOI:** 10.1080/15384047.2024.2342599

**Published:** 2024-04-17

**Authors:** Sean T Evans, Yash Jani, Caroline S Jansen, Ahmet Yildirim, Ecem Kalemoglu, Mehmet Asim Bilen

**Affiliations:** a Department of Medicine, Emory University School of Medicine, Atlanta, GA, USA; b Undergraduate studies, Mercer University, Macon, GA, USA; c Medical Scientist Training Program, Emory University School of Medicine, Atlanta, GA, USA; d Genitourinary Medical Oncology Program, Department of Hematology and Medical Oncology, Winship Cancer Institute of Emory University, Atlanta, GA, USA; e Department of Hematology and Medical Oncology, Emory University School of Medicine, Atlanta, GA, USA; f Department of Biochemistry, Emory University School of Medicine, Atlanta, GA, USA; g Department of Basic Oncology, Health Institute of Ege University, Izmir, Turkey

**Keywords:** Nivolumab, ipilimumab, programmed cell death protein-1 (PD-1), programmed death ligand-1 (PD-L1), immune checkpoint inhibitors, immunotherapy resistance, immunotherapy, renal cell carcinoma, urothelial cell carcinoma, prostate cancer

## Abstract

The introduction of novel immunotherapies has significantly transformed the treatment landscape of genitourinary (GU) cancers, even becoming the standard of care in some settings. One such type of immunotherapy, immune checkpoint inhibitors (ICIs) like nivolumab, ipilimumab, pembrolizumab, and atezolizumab play a pivotal role by disturbing signaling pathways that limit the immune system’s ability to fight tumor cells. Despite the profound impact of these treatments, not all tumors are responsive. Recent research efforts have been focused on understanding how cancer cells manage to evade the immune response and identifying the possible mechanisms behind resistance to immunotherapy. In response, ICIs are being combined with other treatments to reduce resistance and attack cancer cells through multiple cellular pathways. Additionally, novel, targeted strategies are currently being investigated to develop innovative methods of overcoming resistance and treatment failure. This article presents a comprehensive overview of the mechanisms of immunotherapy resistance in GU cancers as currently described in the literature. It explores studies that have identified genetic markers, cytokines, and proteins that may predict resistance or response to immunotherapy. Additionally, we review current efforts to overcome this resistance, which include combination ICIs and sequential therapies, novel insights into the host immune profile, and new targeted therapies. Various approaches that combine immunotherapy with chemotherapy, targeted therapy, vaccines, and radiation have been studied in an effort to more effectively overcome resistance to immunotherapy. While each of these combination therapies has shown some efficacy in clinical trials, a deeper understanding of the immune system’s role underscores the potential of novel targeted therapies as a particularly promising area of current research. Currently, several targeted agents are in development, along with the identification of key immune mediators involved in immunotherapy resistance. Further research is necessary to identify predictors of response.

## ARTICLE HIGHLIGHTS

- Immunotherapy has transformed the treatment landscape for many cancer types, including genitourinary malignancies such as renal and bladder cancers.However, not all patients or tumor types, such as prostate cancer, respond to this type of treatment.
- Understanding the mechanisms of immunotherapy resistance is critical for developing strategies to overcome these challenges.
- Primary resistance, which is present at the onset of treatment, can bedue to genetic abnormalities or immune system dysregulation. These factors alter the interactions between host cells and cancer cells.
- Adaptive resistance develops during therapy due to dynamic changes in the levels of growth factors, cytokines, and the tumor microenvironment (TME).
- Acquired resistance mainly occurs at the genetic and translational levels, involving the downregulation of critical human leukocyte antigen (HLA) molecules and interference with mutational repair.
- Future therapies may focus on detailed genetic profiling of patients to guide treatment selection and on the use of immune profile monitoring to assist in assessing responsiveness, alongside developing novel targeted therapies and ICIs.
- Further research is needed to identify predictors of response to ICIs.

## Introduction

Genitourinary (GU) malignancies, including prostate, testicular, penile, bladder, ureteral, and kidney cancers, represent 24% of new cancer cases, totaling 468,100 new diagnoses annually. Based on the Cancer Statistics Explorer Network SEER*Explorer tool, the 5-year survival rate for bladder cancer at any stage is 77.9%, but this rate drops dramatically to 8.3% for metastatic disease.^1^ A similar trend is observed in prostate cancer, where the 5-year survival rate for any stage of the disease is 97.1%, while the 5-year survival rate for metastatic disease falls to 34.1%.^2^ Despite the annual increase in survival rates of GU cancers thanks to the development of new treatment options, better screening methods, and rapid initiation of therapy, improved treatment strategies remain necessary for metastatic disease in GU cancers.

**Figure 1.**
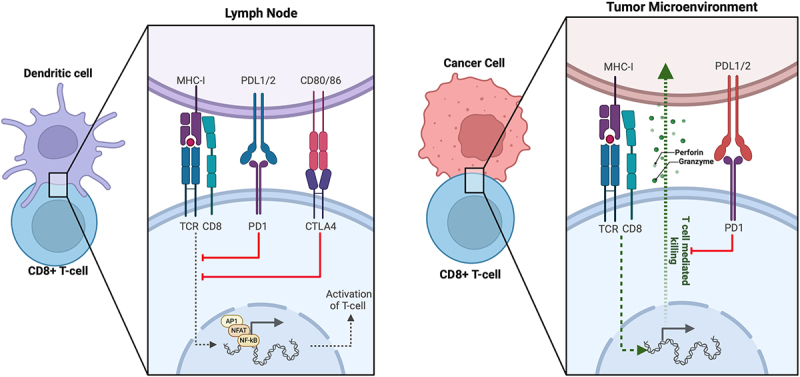
Ligand-receptor interactions involved in immunological recognition of cancer cells.

Immune checkpoint inhibitors (ICIs) were first approved by the FDA in 2011 for the treatment of late-stage melanoma. After being evaluated in numerous clinical trials for the treatment of various cancer types, including GU malignancies, the use of ICIs has shifted the landscape of cancer therapy.^3^ ICIs are monoclonal antibodies that target specific regulatory immune receptors on the surface of cells. The mechanism of action of these agents is based on the inhibition of signals that attenuate the immune response, causing the immune system to exert a more robust immune response against tumor cells. The top three most widely used checkpoint inhibitor classes target immune inhibitory receptors: cytotoxic T lymphocyte-associated molecule-4 (CTLA-4), programmed cell death receptor-1 (PD-1), and programmed cell death receptor ligand-1 (PD-L1).^4^ Figure 1 demonstrates the ligand-receptor interactions involved in immunological recognition of cancer cells on a cellular level. While ICIs have generally demonstrated good tolerability and effectiveness in GU malignancies, a significant number of patients do not respond to therapy, resulting in highly variable outcomes.^5^ In this review, we examine the current mechanisms of resistance to immunotherapy in GU cancers and explore ongoing investigations into novel methods of overcoming this resistance.

## Immunotherapy resistance

Despite the remarkable success of immunotherapy in treating bladder and renal malignancies, not all patients or tumor types respond favorably to these treatments, and many develop resistance. Clinically, this resistance may present as progressive tumor growth, worsening symptoms, or a decline in overall health indicators or functional status. In some cases, resistance can also be monitored through changes in tumor markers, inflammatory markers, immune cell activity, or genetic alternations that can be assessed through laboratory tests and tissue biopsies.^6^ Imaging studies can offer additional visual evidence of resistance by monitoring disease progression or response to therapy. Identifying these indicators is crucial for adapting treatment plans to enhance patient outcomes.

### Primary resistance

Primary resistance can be a formidable barrier to the initial success of immunotherapy treatments. Unlike adaptive or acquired resistance, which develops over time in response to the selective pressure of anti-tumor immunity, primary resistance occurs at the outset of therapy administration. It is rooted in the intrinsic properties of the cancer cells themselves, often manifested through genetic aberrations or dysregulation of factors that typically regulate the immune response. This complex interplay of genetic and micro-environmental factors enables the cancer to withstand immunological attacks.^7^ Exploring the mechanisms of primary resistance uncovers a range of biological factors, from immune pathway disruptions to microbiome effects, that contribute to a cancer’s resistance to immunotherapy.

#### Tumor-intrinsic genetic and genomic factors

Intrinsic tumor factors encompass genetic and molecular alterations within the tumor cells that can inherently impede the efficacy of immunotherapies. Alterations in the mitogen-activated protein kinase (MAPK) pathway may represent a key intrinsic factor contributing to primary resistance in cancer cells. Oncogenic activation of the MAPK pathway is known to lead to the secretion of cytokines such as VEGF and IL-10, known to dampen T-cell activity and recruitment to tumor sites. This has been demonstrated to reduce the efficacy of T-cell-mediated immunotherapies.^8^ Similarly, the loss of a tumor suppressor gene PTEN, which enhances PI3K signaling to promote malignant cell proliferation, is common in many cancers and effectively contributes to resistance against checkpoint inhibitors. The absence of PTEN is associated with reduced expression of immune response factors like IFN-γ (Interferon-gamma) and granzyme-B in immune cells, as well as reduced CD8+ T-cell infiltration. PTEN mutations have also been linked to a higher rate of ICI therapy resistance, suggesting an interplay between these mutations and an immunosuppressive tumor microenvironment (TME).^9^ Alterations of these pathways have also been explored in genitourinary cancers. In renal cell carcinoma (RCC), PTEN mutations, though less common, have been linked to abnormal AKT activation, while MAPK pathway alterations are being explored for their prognostic potential.^10,11^ In bladder cancer, studies have focused on the prevalence and clinical significance of PTEN deletions, suggesting their association with disease progression, though the incidence of PTEN mutations appears low.^12^ On the other hand, prostate cancer presents a higher frequency of PTEN genomic aberrations, seen in up to 20% of primary prostate tumors and 50% of castration-resistant tumors, highlighting their role in disease progression and resistance to therapies.^13^

**Figure 2.**
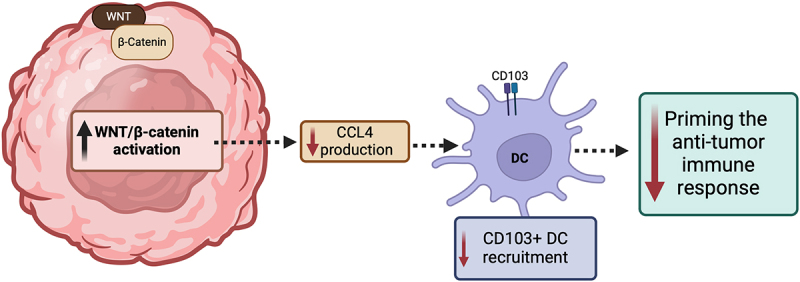
Effect of increased Wnt/β-catenin activation on the tumor response.

The ability of cancer-promoting signaling pathways to prevent T-cells from acting on tumor cells is also seen in the activation of WNT signaling, which is caused by the stabilization of β-catenin. In GU cancers, aberrant WNT signaling, notably in germ cell tumors, prostate, and bladder cancers, has been linked to tumor progression and therapeutic resistance. This emphasizes its potential as a target for novel treatments,^14–17^ Tumors in murine models with elevated β-catenin lacked a particular type of dendritic cells (DCs) known as CD103+ DCs, due to diminished expression of CCL4, a cytokine.^18^ These DCs are particularly important for engaging and activating CD8+ T-cells, given their enhanced ability to efficiently traffic to lymph nodes and cross-present tumor antigen to CD8+ T-cells, priming the anti-tumor immune response.^19,20^ Additionally, ICIs were found to be more effective in targeting tumors with loss of β-catenin compared to those expressing β-catenin.^18^ Similarly, β-catenin signaling pathway-related genes were expressed to a greater degree in non-T-cell inflamed tumors. Studies in patients with melanoma found that individuals who initially responded to anti-PD-1 therapy but then developed metastases (i.e., developed resistance) exhibited new elevations of β-catenin expression and subsequent loss of T-cell infiltration. Mutations that enhance β-catenin signaling were notably prevalent in tumors lacking T-cell inflammation, with the Wnt/β-catenin pathway activated in the majority (90%) of these non-inflamed tumors.^21^ Figure 2 demonstrates the effect of increased Wnt/β-catenin activation on the tumor response.

#### Alterations in antigen processing and presentation

**Figure 3.**
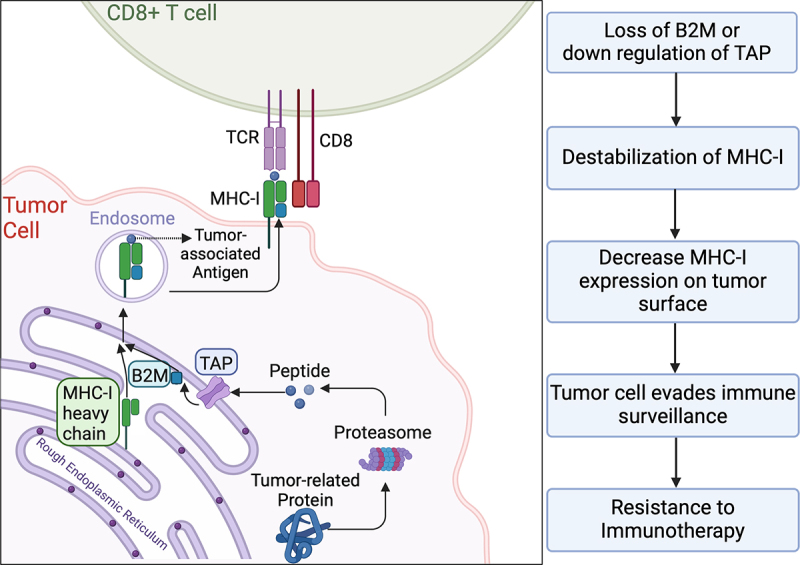
Destabilization of the MHC-I due to loss of B2M.

Studies have also identified the loss of β2-microglobulin (B2M) as a potential molecular pathway of tumor cells to evade immunity. Major histocompatibility complex class I (MHC-I) molecules play a pivotal role in presenting tumor neoantigens to immune cells, and B2M is essential for the proper assembly and presentation of MHC-I molecules on the cell surfaces.^22^ Its absence hinders CD8+ T-cell recognition, impairing a key pathway of immune surveillance.^23^ While B2M loss is somewhat less common in renal cancers compared to bladder and prostate cancers, renal cell carcinomas may exhibit other alterations that affect MHC- I presentation. This highlights the diverse mechanisms of immune evasion in genitourinary tumors. Furthermore, Gettinger et al. found that homozygous loss of B2M leads to the downregulation of MHC-I in cancer cells, further facilitating resistance to ICIs.^24^ Figure 3 demonstrates how loss of B2M destabilizes the MHC-I.

An in vivo experiment demonstrated that immunocompetent mice when treated with anti-PD-1 therapy and injected with B2M-knockout or B2M-wild type cancer cells as a control group. These knockout cancer cells exhibited less sensitivity to PD-1 blockade compared to those in the control group. Further observations indicated a marked decrease in the destructive capability of CD8+ T-cells against these B2M-depleted cancer cells. This suggests that the absence of B2M facilitates evasion from ICIs by altering antigen presentation on the cell surface.^24^ Additionally, comprehensive RNA sequencing, combined with flow cytometric evaluation in human patients with melanoma revealed that lower levels of MHC-I molecules are associated with resistance to PD-1 inhibitors. Interestingly, this resistance was also linked to increased levels of transforming growth factor-β (TGF- β) and cancer-associated fibroblast (CAF) signatures.^22^

#### Microbiome-mediated effects

Recent findings have underscored the significant influence of the microbiome and intratumoral microbes on anti-tumor immune responses and the effectiveness of ICIs. Multiple retrospective studies have provided evidence linking the gut microbiota with prostate cancer, showing an increased incidence of prostate cancer in patients with inflammatory bowel disease (IBD) compared to matched controls,^25–27^ In a study with 20 male participants, a higher presence of Bacteroides massiliensis was observed in the gastrointestinal microbiota of those diagnosed with prostate cancer while individuals without the disease exhibited a greater prevalence of Faecalibacterium prausnitzii and Eubacterium rectale. Notably, F. prausnitzii, a member of the Firmicutes phylum, is recognized for its production of butyrate, a short-chain fatty acid that exhibits anti-inflammatory effects, particularly in cases of colitis. In another study, researchers compared the gut microbiota of mice with prostate tumors, created by injecting a specific human metastatic cancer cell line (CRPC 22Rv1-M4), and healthy mice. The findings showed that mice with prostate tumors exhibited a higher concentration of certain bacterial families, specifically Akkermansiaceae, Bifidobacteriaceae, and Enterococcaceae. Furthermore, the study noted an increase in bacterial groups known for their steroid-producing ability in the mice injected with prostate cancer cells. This increase was associated with a more rapid progression of the disease.^28^

Research has displayed the impact of prostate cancer-specific therapies, such as androgen receptor-targeted therapy (ATT), on gastrointestinal microbes. These microbes are not only affected by such treatments but also contribute to the development of resistance to these therapies. In patients with castration-resistant prostate cancer, gut microbes have the capability to convert pregnenolone into androgenic steroids. This has been demonstrated in mouse models, which have shown that androgen deprivation therapy leads to significant changes in the composition of the gut microbiome. This includes an increased presence of Ruminococcus and Bacteroides species, particularly observed in mice with castration-resistant disease.^29^ This finding has been further corroborated through comprehensive metagenomic sequences in human cohorts of both hormone-sensitive and castration-resistant prostate cancer patients. A similar study examining the stool microbiota of 30 individuals across various stages of prostate cancer identified an elevated presence of bacteria involved in steroid biosynthesis within the gut microbiota of patients undergoing androgen-targeted treatments, compared to those not receiving any treatment. Species such as A. muciniphila, Ruminococcaceae, and Lachnospiraceae spp. were notably more prevalent in the stool samples of patients undergoing oral ATT.^30^

Various studies have identified a diverse array of microbes within different types of cancers in addition to prostate cancers. These microbes are believed to play a role in cancer progression, from initiation to metastasis, and may even influence patient survival outcomes. For instance, in pancreatic cancer, certain microbial profiles have been correlated with longer survival.^31^ In preclinical models, responses to therapies such as anti-CTLA-4 were found to be dependent on bacterial species, as mice that were germ-free or antibiotic-treated did not respond to the therapy.^32^ These findings were further supported by clinical reports indicating that patients treated with ICIs who also received antibiotics experienced decreased overall survival (OS) and progression-free survival (PFS). Similar findings were observed for anti-PD-1, where other microbes were also implicated in influencing the therapy’s effectiveness.^33^ It has been suggested that tumor cells may have the ability to present peptides derived from internal bacteria on their surface using human MHC molecules. This process potentially allows them to be recognized by T-cells that infiltrate the tumor. This mechanism might be a pathway through which bacteria present in tumors directly modulate the body’s immune defense against cancer.^34^ In the context of RCC, studies highlighted the gut microbiota’s significant role in enhancing clinical responses to ICIs.^35,36^ For instance, De Rosa et al. observed in patients with advanced RCC that a diminished response to treatment was associated with the abundance of certain bacteria types such as Erysipelotrichaceae bacterium and Clostridium hathewayi.^37^

These studies have expanded their scope to include bladder and prostate cancer, revealing that gut microbiome composition influences PD-1 blockade efficacy. In prostate cancer, recent literature highlights the microbiota’s role in the disease’s pathogenesis and treatment strategies. It has been indicated that specific species within the urinary and gut microbiome are associated with an increased risk of developing prostate cancer and its subsequent progression.^38^ This evidence underscores the critical role of the microbiota in carcinogenesis, suggesting that modulating the host microbiota could offer new avenues for improving early detection rates and developing new treatment strategies for prostate cancer.^39^

#### PD-L1 mediated resistance

In addition to tumor genetic factors, immunological factors further complicate the landscape of primary resistance. The expression of PD-L1, a protein commonly found on the surface of cancer cells, plays a crucial role in enabling tumors to evade the immune system. This mechanism is also positively associated with response to ICIs across various types of cancers.^40^ Tumors that express PD-L1 can bind to PD-1 receptors on T-cells, thereby facilitating immune system evasion by leveraging this natural negative feedback mechanism design to dampen T-cell activation and activity. Given its canonical role in negatively regulating an activated immune system, the presence of PD-L1 may also indicate an attenuated, yet activated, immune microenvironment.^41^ Consequently, tumors with low or no expression of PD-L1 may tend to be resistant to anti-PD-1 and anti-PD-L1 treatments, as seen in breast and lung cancers which have historically demonstrated low PD-L1 expression.^42,43^ Despite this trend, the absence of PD-L1 does not necessarily preclude a positive response to such therapies, nor does its presence guarantee the treatment success. This suggests that the efficacy of immunotherapy is determined by a broader spectrum of factors beyond just PD-L1 expression.^40^

#### Influence of tumor mutational burden

Tumor mutational burden (TMB) is another well-studied potential biomarker, that predicts the efficacy of immunotherapies, such as PD-1 and CTLA-4 inhibitors, across a spectrum of cancers. This metric quantifies the number of mutations within a megabase of tumor DNA and has shown a significant correlation with treatment success in certain tumor types.^44^ For example, lung cancer patients with higher TMB levels experienced better outcomes with nivolumab, as shown by exome analyzes.^45^ Furthermore, the KEYNOTE-158 study demonstrated that a high TMB contributed to the effectiveness of pembrolizumab, leading to its FDA approval for TMB-high tumors. Despite the strong link between TMB and treatment response, tumors with lower mutational loads, such as those found in prostate and pancreatic cancers, typically exhibit less favorable responses to immunotherapies.^45^ Nevertheless, certain studies point out exceptions, showing that low TMB does not universally predict poor outcomes.^46^ In cases like metastatic renal cell carcinoma and polyomavirus-positive Merkel cell carcinoma, patients have still responded positively to ICIs despite their low TMB.^47^ Thus, although a high TMB is generally associated with better response to ICIs, the interplay between mutational burden and immunotherapy efficacy is complex and influenced by factors beyond TMB alone.

## Adaptive resistance

Adaptive resistance primarily involves dynamic tumor modifications, whereby tumors counteract the immune system’s attacks during therapy. This phenomenon not only complicates treatment strategies but also underscores the urgent need for innovative approaches to overcome or prevent tumor adaptations and developed resistance to current therapies. For example, dynamic expression of vascular endothelial growth factor (VEGF) and interleukin-8 (IL-8) by tumor cells have been identified in several preclinical models and some human cancers as another strategy to modulate the TME in favor of tumor survival and cancer cell proliferation.^48^ VEGF plays a particularly crucial role in renal cancers, making it a common target for therapeutic strategies. In RCC, its role in promoting angiogenesis is pivotal, as it not only sustains tumor growth but also contributes to creating an immunosuppressive microenvironment, a hallmark of this cancer type’s progression.^49^ Angiogenesis, the formation of new blood vessels, is essential for supplying the tumor with necessary nutrients and oxygen. Moreover, it enables the tumor to exert its influence on the immune system, further emphasizing the multifaceted impact of VEGF in cancer dynamics.

VEGF is also known to contribute to the creation of an immunosuppressive environment.^50^ It directly inhibits the maturation and function of dendritic cells, crucial orchestrators of the immune response, thereby impairing the T-cell activation.^51^ Furthermore, VEGF facilitates the recruitment of regulatory T-cells (Tregs) and myeloid-derived suppressor cells (MDSCs), both essential in maintaining the immune suppressive TME.^52^ By promoting these suppressive cell populations, VEGF helps shield the tumor from immune surveillance. RCC, especially its most common form, clear cell renal cell carcinoma (ccRCC), the VEGF pathway plays a crucial role due to the inactivation of the von Hippel-Lindau (VHL) tumor suppressor gene. This leads to the upregulation of hypoxia-inducible factors (HIF), which in turn drive VEGF overexpression, a key factor in the progression of RCC.^53^

IL-8 works synergistically with VEGF to create an immunosuppressive TME. Acting as a chemokine, it attracts neutrophils and MDSCs to the tumor site. These cells then release proteases and reactive oxygen species, creating a hostile environment that inhibits T-cell function and thus further impairs the anti-tumor immune response.^54^ Additionally, IL-8 is implicated in enhancing tumor invasiveness and metastasis by promoting the epithelial-mesenchymal transition (EMT) of tumor cells, which facilitates their spread throughout the body.^55^ Together, VEGF and IL-8 orchestrate a microenvironment that not only favors tumor growth and spread but also renders strategically less accessible to immune effector cells. Specifically, in the context of GU tumors, the role of IL-8 is crucial as it contributes to a TME that facilitates tumor progression and metastasis. This is evidenced by elevated IL-8 expression observed not only in various tumor cell lines and tissues but also in the peripheral blood of cancer patients. Specifically, the interaction of IL-8 with components of the TME, such as fibroblasts, endothelial cells, and immune cells, plays a significant in molding the immune landscape within GU tumors. This interaction affects both tumor progression and therapeutic responses. Moreover, elevated IL-8 expression leads to the recruitment of myeloid cell lines including MDSCs, CD15+ monocytes, and neutrophils, which have been shown to suppress adaptive T-cell antitumor immunity,^56–58^ Transcriptomic analysis of circulating and tumor-infiltrating IL-8-producing MDSCs has revealed an increased expression of pro-inflammatory myeloid genes coupled with downregulation of antigen-presentation and interferon-inducible genes, further underscoring the role of IL-8 in compromising adaptive immunity in the context of the GU tumors.^59^

Interferon-gamma (IFN-γ) plays a dual-faceted role in tumor progression and immune surveillance. Produced by CD8+ T-cells, IFN-γ enhances antigen presentation, facilitates immune cell recruitment, and exerts anti-proliferative effects on tumors. However, continuous exposure to this factor can lead to genetic instability in tumors, followed by altered DNA repair and editing genes. This genetic reshuffling may result in tumor escape from immune detection and the development of resistance.^60^ Central to this resistance mechanism is the mutation or downregulation of components in the IFN-γ signaling pathway, particularly the JAK-STAT signaling cascade. Specific mutations, such as those in the IFN-γ receptor kinases JAK1 and JAK2, are associated with resistance to immunotherapies, including anti-CTLA-4 and anti-PD-1 treatments, which may be mediated through decreased sensitivity to IFN-γ. For instance, tumors harboring these mutations often exhibit a lack of PD-L1 expression upon IFN-γ exposure, making them less susceptible to T-cell-mediated destruction.^61^ IFN-γ plays a pivotal role in tumor immunity by enhancing antigen presentation, facilitating immune cell recruitment, and exerting anti-proliferative effects on tumors. It inhibits angiogenesis in tumor tissue and induces apoptosis in Tregs, as well as stimulates the activity of M1 proinflammatory macrophages, which are all crucial for combating tumor progression. Its pleiotropic nature, encompassing both antiviral, antitumor, and immunomodulatory functions, allows IFN-γ to coordinate both innate and adaptive immune responses. However, understanding this complex dual role in promoting pro-tumorigenic and antitumor immunity within the TME is critical, especially in the context of GU tumors.^62^

The TME plays a major role in enabling tumor immune evasion and resistance to immunotherapy, primarily through the secretion of various immunomodulatory substances. In the context of immunotherapy, outcomes are significantly influenced by factors such as the decline in immune function associated with aging, overall tumor mutational burden, and mutations in specific tumor suppressor/DNA damage repair genes. Among these, midkine, a protein linked with negative outcomes and decreased responsiveness to therapies targeting the PD-1/PD-L1 axis in melanoma, has also been identified in the TME of GU tumors. High midkine expression correlates with a TME that supports the presence of Tregs and tumor-associated macrophages (TAMs), both of which contribute to tumor progression and are linked to a poorer prognosis.^63^

In RCC, alterations in the VHL gene cause abnormal HIF accumulation and the activation of angiogenic pathways, affecting both disease progression and severity. The TME in RCC, characterized by inflammation and the presence of immune cells, necessitates a shift in treatment focus toward modifying the microenvironment rather than directly targeting the tumor cells. Prostate adenocarcinoma, the primary treatment approach involves hormone-based therapies such as chemical castration or androgen deprivation therapy (ADT). However, resistance to ADT often necessitates combined therapeutic approaches, influenced by the complex cellular interactions within the TME. In bladder cancer, the variability in the immune profile of its TME further complicates the prediction of immunotherapy efficacy.^64^

In the context of cytokines within the TME, TGF-β emerges as a multifaceted regulator, affecting a wide range of cellular interactions. Elevated levels of TGF-β have been associated with reduced effectiveness of anti-PD-1 therapy and overall poorer patient outcomes.^65^ Moreover, the influence of TGF-β’s on stromal components, such as cancer-associated fibroblasts, has been linked to decreased T-cell infiltration, which potentially leads to an environment that excludes these vital anti-tumor immune cells.^66^ Adenosine, another immune-modulating agent found within the TME, has been observed to contribute to creating an immunosuppressive environment in GU cancers that adversely affecting patient prognosis.^67^ Clinical trials are currently investigating new therapies that target adenosine pathways alongside anti-PD-1 treatment.^65^ Despite progress in understanding these dynamic immune components within the TME of GU cancers, significant knowledge gaps persist. A deeper understanding of these mechanisms holds the potential for the development of prognostic and predictive biomarkers, thereby increasing the precision of immunotherapy and improving therapeutic outcomes in GU cancers.

In addition to the tumor-immune cell interactions within the TME, metabolic dynamics, such as hypoxia, play a crucial role in influencing tumor immunity. Hypoxia is one of the hallmarks of the TME, promoting unregulated cell proliferation in tumors and erratic vascular growth, thus limiting essential resources like oxygen. These hypoxic conditions within tumors lead to diminished MHC-I expression in tumor cells and dendritic cells.^68^ Additionally, within the RCC TME, tumor-associated immune cells including T-cells and NK cells, often exhibit compromised mitochondrial function.^69^ Notably, hypoxia promotes the secretion of T-cell suppressors like adenosine and galectin-1. While adenosine increases intracellular cAMP levels which leads to immune suppression, galectin-1 plays a crucial role in cell adherence, tissue invasion, and vascular formation.^70,71^ Furthermore, hypoxia within the TME attracts TAMs to these hypoxic zones. TAMs, in turn, are known to increase resistance to several cancer treatments and elevate the risk of cancer recurrence.^72^

**Figure 4.**
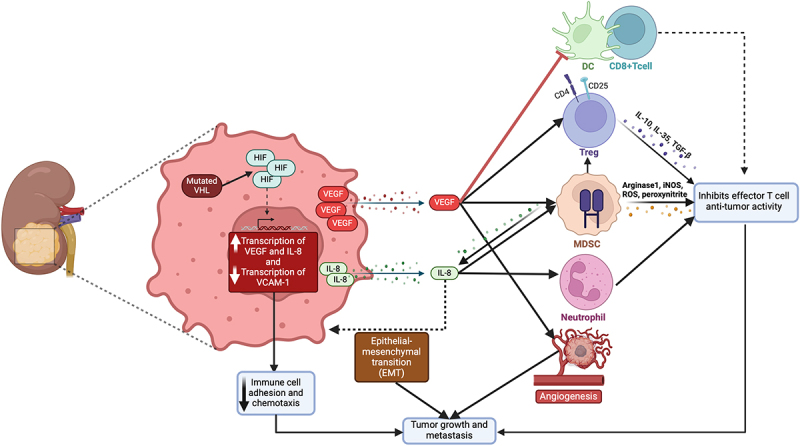
Down-stream effects of VHL dysfunction.

Mutations in the VHL gene, commonly seen in ccRCC, impair the function of the VHL protein. These mutations are crucial in the development of ccRCC by altering the cell’s response to hypoxia, which is critical for regulating angiogenesis, cell proliferation, and tumor progression.^73^ The VHL gene is key in regulating HIFs, including HIF1α and HIF2α. Figure 4 depicts the down-stream effects of VHL dysfunction. Normally, VHL mediates the breakdown of HIFα subunits under oxygen-rich conditions. However, with dysfunctional VHL, as often occurs in ccRCC, there’s continuous activation of HIF-1, which promotes the angiogenic nature of these tumors.^74^ Furthermore, a study investigating the role of VHL in ccRCC found that the loss of VHL, the presence of hypoxia, or the inactivation of prolyl hydroxylase domain-containing proteins reduces vascular cell adhesion molecule 1 (VCAM-1) levels through a transcriptional mechanism. VCAM-1 is essential for immune cell adhesion and chemotaxis, and its reduced expression is linked to tumor aggression. Interestingly, in ccRCC samples with nonmissense VHL mutations, there’s an inverse correlation between VCAM-1 level and indicators of tumor aggressiveness such as tumor stage, symptoms, and microvascular invasion, suggesting that higher VCAM-1 level, with functional VHL protein, might enhance antitumor immunity.^75^ These insights underscore the significant role of VHL alterations in affecting immune cell function and the tumor microenvironment, leading to a TME that is suppressive to the immune system and presents obstacles to successful immunotherapy in RCC.

In a study conducted by Brauch et al. on 227 sporadic ccRCC tumors analyzed for VHL-altering events, it was found that the combined rate of VHL mutation and promoter hypermethylation was 45%.^75^ The study further identified that the presence of VHL mutation or hypermutation events was significantly associated with poor prognostic markers, notably for the pT3 tumor stage. Another study examining 187 patients with ccRCC revealed that VHL mutations were associated with worse patient outcomes at Stage III. However, in Stage IV cases, this correlation was not significant. This suggests that the impact of VHL mutations is more pronounced in earlier stages when assessing the potential for metastatic disease.^76^ This highlights the complexity and adaptability of RCC tumors in evading immune surveillance, especially considering the near-universal presence of alterations in hypoxia sensing mechanisms, such as VHL mutations, in these tumors.

CAFs play a pivotal role in shaping the TME of GU cancers, particularly in terms of the immunosuppressive features of that environment. These cells, found in both primary and metastatic tumors, are involved in a range of functions, including extracellular matrix remodeling, metabolism modulation, angiogenesis, and the production of growth factors, cytokines, and chemokines.^77^ They display a high degree of plasticity and versatility, adapting and responding to the dynamic TME.^78^ The interactions between the stroma and tumor cells are related to resistance to ICIs. CAFs are implicated in creating an environment that impedes T-cell activity, contributing to the formation of “tumor deserts” that exclude immune cells. Furthermore, the patterns of gene expression related to CAFs are correlated with reduced T-cell infiltration and diminished responsiveness to widely used ICIs such as nivolumab.^79^

The extracellular matrix built by CAFs limits T-cell movement, potentially hindering their tumor-killing functions. It has been reported that substances secreted by CAFs can weaken immune defenses against the tumor.^80^ A study focusing on the role of CAFs in bladder cancer progression found that CAFs, activated by cancer-derived exosomes, induce EMT in cancer cells, thereby enhancing their growth, migration, and invasion. This process was primarily driven by the cytokine IL-6 secreted by CAFs, with the inhibition of IL-6 significantly reversing the EMT phenotype, highlighting its crucial role in bladder cancer progression.^81^ In another study examining the impact of CAFs in urothelial bladder cancer (UBC), it was revealed that CAFs, through the overexpression of miR-146a-5p, promote cancer stemness and chemoresistance to drugs like gemcitabine and cisplatin. This process involves miR-146a-5p’s regulation of key signaling pathways in UBC cells, where increased levels of this microRNA in patient serum are correlated with advanced tumor stages and increased relapse risk, suggesting its potential as both a biomarker and therapeutic target in UBC.^82^

Beyond CAFs, other stromal cells specific to certain tissues can also play a role in influencing resistance to ICIs. In the case of prostate cancer bone metastases, resistance to ICIs has been partially attributed to the release of TGF-β following bone degradation, which affects the numbers of T helper type 1 (Th1) cells.^83^ Additionally, in brain metastases, phosphoSTAT3-positive astrocytes have been shown to suppress CD8+ T-cell activity and increase a subset of microglia/macrophages that facilitate immune evasion, potentially contributing to immunotherapy resistance.^84^ In the context of GU cancers, these insights into the biology and function of CAFs are essential for developing more effective therapeutic strategies. The identification and precise characterization of different populations of CAFs and their tumor-promoting or restraining functions could lead to novel diagnostic and therapeutic approaches. As our understanding of the diversity and roles of these CAFs in cancer evolves, it offers new avenues for targeted therapies that could potentially improve outcomes in GU cancers.

## Acquired resistance

In the context of acquired resistance, one of the broad categories of escape strategies that impact the response to anti-PD-1 therapies is the loss of heterozygosity in HLA genes. These genes are essential for distinguishing self from non-self and clearing pathogens. Notably, 40% of patients exhibiting loss of HLA heterozygosity also showed a consequent loss of neoantigen presentation, leading to immune evasion.^85^ Moreover, cancer cells have developed multiple strategies for HLA downregulation, including genetic, epigenetic, post-transcriptional, and translational modifications, all aimed at evading detection by the immune system.^86^ For example, in a case of metastatic melanoma, an initially effective response to pembrolizumab, an anti-PD-1 therapy, was eventually overcome by resistance due to a new truncating mutation in the B2M gene, crucial for the stability of MHC-I.^87^ Further studies have reinforced the important role of these genetic factors in immune response, revealing that mismatch repair-deficient tumors that are resistant to anti-PD-1 therapy also contain mutations in B2M gene.^88^

Epigenetic modifications have also been shown to play a role in resistance against ICIs. Alterations in the epigenetic landscape of tumor cells, such as those involving the Switch/Sucrose non-fermenting (SWI-SNF) chromatin remodeling complex, have been implicated in sensitizing tumors to ICIs. Epigenetic modulators can enhance the anti-tumor response of ICIs by increasing the production of chemokines crucial for attracting CD8+ T-cells to the tumor microenvironment.^89^ Despite these findings, the exact role of the tumor’s epigenetic makeup in resistance, particularly in relation to T-cell exhaustion, remains unclear, necessitating more research in this area.^90^ Mutations and epigenetic silencing within the interferon receptor signaling pathway have been shown to negate the anti-tumor effects of IFN-γ, demonstrating how the tumor can escape immune surveillance. These alterations can lead to diminished responses to the potent anti-cancer activity of IFN-γ.^91^ Furthermore, genetic and epigenetic alterations that impair antigen presentation are also known to contribute to both primary and acquired resistance to ICIs, an effect observed irrespective of the TMB.^92^ This highlights the complexity of resistance mechanisms, suggesting that a high TMB alone is not sufficient to ensure responsiveness to ICIs, as cancer cells can evade the immune system by reducing MHC expression through a combination of genetic and epigenetic mechanisms. These mechanisms include NF-kB pathway dysregulation, hypermethylation and deacetylation of the MHC promoter, interference by noncoding microRNAs, and suppression of chaperone protein expression, all contributing to reduced antigen presentation and immune detection.^93,94^

Recent epigenetic investigations of RCC have highlighted the significant role of Polybromo-1 (PBRM1) gene mutations in creating a TME that is less responsive to immune system interventions. Specifically, the loss of PBRM1 disrupts the interaction between the Brahma-related gene 1 (BRG1) and the IFN-γ receptor 2 (IFN-γ r2) promoter, leading to decreased STAT1 activation and IFN-γ target gene expression. This impacts IFNγ-STAT1 signaling pathways in both human and murine RCC cell lines, resulting in a decrease in immune response within the tumor and manifesting as resistance to ICIs. This resistance was parallel in clinical observations of nearly 700 ccRCC patients, showing poor response to ICIs and reduced immune cell infiltration in cases with PBRM1 mutations. Further research into human ccRCC has revealed a significant link between PBRM1 mutations and reduced expression of genes within the immunomodulatory signature. This pattern, affecting immune responses like IFN-γ and IFN-α, was consistent across various patient cohorts, reinforcing the notion that PBRM1 mutations on the immune dynamics of RCC and its responsiveness to immunotherapy.^95^ These mutations not only disrupt crucial immune signaling pathways but also alter the tumor’s immune environment, influencing the tumor’s response to immunotherapy.

Tregs, characterized by expression of CD4, CD25, and the transcription factor FoxP3, play a pivotal role in immune regulation within the TME. They help maintain self-tolerance by restricting the activity of CD4+ and CD8+ T-cells through various mechanisms, including the secretion of immunosuppressive cytokines such as IL-2, IL-10, IL-35, and TGF-β.^96^ The presence of Tregs is associated with a dampening of the anti-tumor response, and the ratio of effector T-cells (Teff) to Tregs has been suggested as a predicting factor for the efficacy of ICIs.^97^ An increased Teff-to-Treg ratio has been correlated with improved responses to these treatments. Conversely, resistance to ICIs may develop due to compensatory Treg proliferation or the upregulation of alternative checkpoint molecules on these cells.^98^ A study involving bladder cancer patients who underwent radical cystectomy revealed a higher frequency of CD4+ Tregs, identified by high expression of CD4, CD25, FOXP3, and low/neg CD127 expression, in tumor-draining lymph nodes in cases of advanced-stage disease.^99^ These findings suggest that Tregs may hinder anti-tumor immune responses, highlighting their potential impact on the effectiveness of immunotherapy in bladder cancer treatment.

MDSCs have been identified as key players in creating an immunosuppressive environment in the TME. They impair the functions of Teff and NK cells through mechanisms involving substances such as arginase 1, inducible nitric oxide synthase (iNOS), reactive oxygen species (ROS), and peroxynitrite.^100^ Furthermore, MDSCs influence the maturation of Tregs and drive macrophages toward a suppressive phenotype, contributing to tumor angiogenesis, invasion, and metastasis.^101^ Clinical data indicate a correlation between MDSCs presence and poorer cancer outcomes, including diminished responses to immunotherapies.^102^

Experimental studies in rodent cancer models have revealed that inhibiting macrophage PI3Kγ activity enhances the response to ICIs, reduces tumor size, and improves survival.^103^ This improvement is associated with heightened pro-inflammatory signaling and decreased suppressive factors within tumors. Furthermore, inhibiting PI3Kγ leads to the upregulation of immune activation markers in macrophages, pinpointing PI3Kγ as a pivotal regulator of macrophage function in cancer.^104^

Investigations into the dynamic nature of TAMs have revealed a wide range of functional states, notably the classic pro-inflammatory, tumor-combative M1 macrophages and the anti-inflammatory, tumor-promoting M2 macrophages, which serve opposing roles. M1 macrophages are known for their role in activating anti-tumor immunity through inflammatory cytokines, whereas M2 macrophages are implicated in down-regulating immune responses by releasing cytokines that suppress CD8+ T-cell activity, recruit Tregs, and facilitate the evasion of tumor cells from immune surveillance. M2 macrophages also often exhibit surface molecules like PD-L1 which further contribute to their ability to suppress immune responses.^105,106^ Various studies have shown that disrupting the M2 macrophage pathway and guiding these cells toward an M1 phenotype can potentially improve the efficacy of ICIs.^107^ In addition, an imbalance marked by a lower ratio of adaptive immune responses to pro-tumorigenic signals within phagocytic myeloid cells has been associated with a lack of response to PD-L1 blockade therapy, particularly in cancer types such as urothelial cancer.^108^

In solid tumors, such as prostate cancer, a significant portion of the tumor mass consists of macrophages, particularly TAMs, which predominantly exhibit M2-like characteristics. These TAMs play a crucial role in various cancer stages, from the initial development to metastasis, by influencing the tumor’s malignant properties. A higher presence of TAMs in prostate cancer correlates with more severe clinicopathological features, making them a key focus for new treatments. Specifically, in advanced stages like metastatic castration-resistant prostate cancer, an increase in M2-TAMs is linked to a higher likelihood of cancer recurrence and increased mortality rates. This trend highlights the pivotal role of macrophage behavior in determining cancer outcomes. Notably, the prevalence of M2-TAMs progressively increases from healthy prostate tissue to high-stage prostate cancer, and their high concentration is closely associated with elevated Gleason scores, indicating poorer survival rates and a higher chance of recurrence following hormone therapy.^109^ This emerging evidence underscores the significance of macrophage plasticity in influencing the outcomes of immunotherapies.

Within the spectrum of acquired resistance, genetic and cellular changes in cancer cells are particularly critical, as they can profoundly alter the tumor’s interaction with therapeutic agents and the immune system. Cancer cells can evade immune detection and become resistant to treatments like ICIs by acquiring mutations that alter the antigens targeted by immune cells.^110,111^ As mentioned above, mutations affecting the IFN-γ pathway, the aging-associated immune function decline, the overall tumor mutational burden, and mutations in specific tumor suppressor/DNA damage repair genes (KDM6A, PTEN, STAG2, TP53, ATM, and BRCA2) have been associated with suboptimal responses in GU cancers.^112,113^ Additionally, the behavior of TAMs and host CD8+ T-cells is crucial to the effectiveness of immunotherapy. Further investigation into epigenetics and the cellular interactions between immune and tumor cells is crucial to tailoring current treatments and identifying novel therapeutic targets.

## Approaches to predicting and overcoming immunotherapy resistance

**Figure 5.**
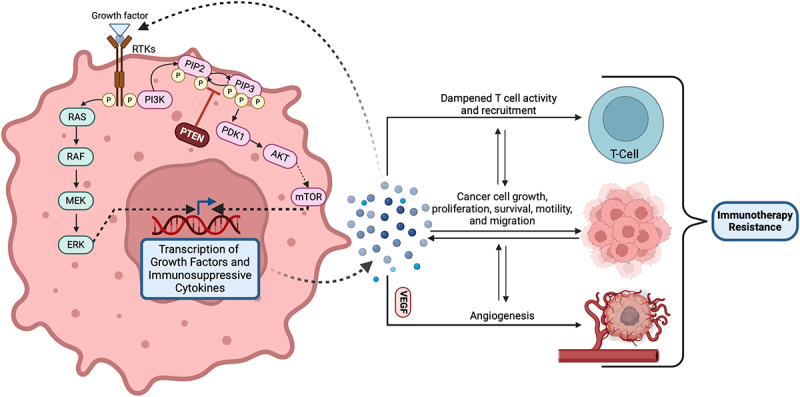
Role of PTEN within the tumor microenvironment.

As discussed above, primary resistance involves patient factors that are present and often quantified prior to initiating therapy. One such widely utilized example is the percentage of PD-L1 expression on tumor cells, intended to predict the efficacy of PD-L1 inhibition, though outcomes vary. Additional pathways, proteins, and cytokines of interest for predicting the efficacy of immunotherapies include MAPK, PTEN, WNT, IL-8, and IFN. Zhang, et. al. recently discussed a prognostic risk model of MAPK pathway-related proteins in ccRCC.^114^ Using RNA sequencing data and transcriptome analyzes, they identified numerous MAPK-related genes with abnormal expression in ccRCC patients and subsequently were able to risk-stratify patients and identify those patients at high risk for severe and progressive disease based on their data. An additional study in urothelial carcinoma by Bekele, et. al. demonstrated that RAF1 amplification may increase susceptibility to MAPK-directed treatments.^115^ Liu et. al. investigated the loss of PTEN association with therapeutics, finding that PTEN-deficient cells demonstrated hypersensitivity to rapalogs such as everolimus and temsirolimus.^116^ Figure 5 demonstrates the role of PTEN in the TME. This suggests the importance of expanding pre-treatment genetic and biomarker profiling to identify alterations that may influence therapy responsiveness. Genetic profiling could also uncover susceptibilities to combination therapies that are not previously considered.

In the context of cytokines, several trials, including analyzes from the CheckMate025 and IMMotion150 trial cohorts that analyzed RCC patient samples following treatment with ICIs (both combination or single agent), reported poorer outcomes for individuals with higher pre-treatment plasma levels of IL-8. Elevated initial plasma IL-8 levels may refine patient selection for ICIs, particularly for those with a high T-cell effector signature score. This score reflects CD8+ T-cell activity and is associated with improved outcomes of ICIs in RCC and urothelial cancer. Patients with both a high T-cell effector score and low IL-8 levels tend to benefit the most from ICIs.^48^

IFN-γ has additionally been explored in the context of GU tumors. Sakatani et al.‘s study on advanced urothelial cancer patients found that high IFN-γ expression in the TME significantly correlates with improved PFS following pembrolizumab treatment, highlighting IFN-γ’s prognostic relevance in response to ICIs.^117^ In addition, a study involving 234 urinary bladder cancer patients demonstrated that a high IFN-γ gene signature is inversely related to mortality risk.^118^ Particularly, those with the highest IFN-γ gene signature tertile had a substantially reduced mortality risk, indicative of better survival outcomes. These insights into IFN-γ’s impact on GU tumors emphasize the importance of understanding its complex role in tumor progression and immunotherapy response, including its interaction with key pathways like the JAK-STAT signaling cascade.^62^

As previously mentioned, the gut microbiome has been studied to identify baseline characteristics that might identify patients as either more or less susceptible to ICIs. A phase I trial involving nivolumab and ipilimumab, with or without the live bacterial supplement CBM588 (bacterium spp Clostridium butyricum), showed a trend toward improved outcomes. This suggests that manipulation of the gut microbiome could enhance the response to ICIs.^119^ Similarly, a randomized trial found that dietary interventions with a probiotic containing Bifidobacterium spp. in patients receiving VEGF tyrosine kinase inhibitors (VEGF-TKIs) successfully modulated the microbiota levels of this bacteria and led to some clinical benefit in the treatment group.^120^ In RCC patients receiving ICIs, greater gut microbiome diversity was associated with more positive treatment outcomes and heightened clinical benefit.^121^

Immune profiling of patients may also hold significance in the context of previously discussed immunomodulation across various cancer types. For example, studies involving ovarian cancer have shown that an increased presence of Tregs is often associated with unfavorable prognoses.^122^ Current research is exploring strategies such as reducing Treg numbers and modifying checkpoints with antibodies to enhance the presence of tumor-reactive tumor-infiltrating lymphocytes (TILs), thereby potentially inhibiting cancer progression. The therapeutic goal is to shift the balance toward Teff, thereby amplifying the immune system’s ability to target and destroy tumor cells. Interestingly, a high baseline level of FoxP3+ Tregs within a tumor does not necessarily predict poor outcomes.^98,123^ In some cases, these Tregs co-exist with active immune responses, and their presence has been associated with improved clinical outcomes following anti-CTLA-4 therapy.^123^ Ongoing research is focused on fully understanding the role of Tregs and their impact on the efficacy of immunotherapies in different cancer settings.

In a similar vein, the concurrent administration of a PI3Kγ inhibitor with anti-PD-1 therapy significantly elevated the success rate of tumor rejection and increased survival in animal studies related to bladder cancer.^104^ Investigators analyzed MDSCs in 113 bladder cancer patients and found that these patients had higher numbers of MDSCs compared to healthy volunteers, with MDSC counts correlating with clinical grade, stage, and poor prognosis.^124^ The study also revealed that lower serum IL-6 levels in bladder cancer patients were associated with increased MDSC formation, suggesting that targeting IL-6 signaling could be a potential treatment strategy. This highlights MDSCs as a possible prognostic marker in bladder cancer.

An additional study focusing on the role of MDSCs in cisplatin-resistant bladder cancer revealed that these cells, particularly the monocytic subtype, are more prevalent in resistant tumors. It was demonstrated that therapies targeting MDSCs, when used in combination with PD-L1 inhibitors, significantly reduced tumor size and increased CD + 8 T-cell activity, suggesting a promising approach for treating cisplatin-resistant bladder cancers.^125^ Furthermore, a systematic review and meta-analysis, which included 16 studies with 1864 patients, found that a higher frequency of MDSCs was associated with shorter OS and poor disease-free survival/recurrence-free survival after treatment.^126^

Therapeutic approaches such as Chimeric antigen receptor T (CAR T) cells and Bi-specific T cell engagers (BiTE) have been developed as an option for resistant malignancies associated with minimal response to ICIs. These more novel agents utilize the patient’s own immune cells which are extracted and bioengineered to identify, bind to, and eliminate cancer cells more effectively.^127^ Most relevant to our discussion in this review are the currently described methods of resistance observed in CAR T and BiTE therapies. Shen et al. describe how loss of CD58 influences resistance via down-regulation of the extrinsic apoptotic pathway, and how inhibiting PI3K expression may potentiate the efficacy of BiTE therapy.^128^ Interestingly, the group also demonstrated that loss of IFN-γ signaling, while typically associated with resistance to ICIs, was associated with greater efficacy of BiTE therapy.^128,129^ This relationship demonstrates the dynamic role of IFN-γ signaling in T-cell based therapies. Studies have also identified that co-expression of CCR8 and TGF-β receptor 2 on CAR T cells improves their ability to overcome innate immune suppression by T regulatory cells.^130^ Currently, these treatments are reserved for patients who have undergone multiple lines of chemotherapy and demonstrated treatment failure, and as such further research is needed to study the impact of chemotherapy-driven immune alteration on the efficacy of CAR T and BiTE therapy, as well as the contextual role of the TME on the modality of treatment.

## Conclusion

Immunotherapy has proven its effectiveness in treating various GU malignancies, particularly in bladder and kidney cancers. Despite the increasing use of ICIs in combination with each other, chemotherapy, and other targeted therapies, a significant number of patients still experience progression on treatment. As discussed, there are multiple mechanisms of immunotherapy resistance that require further investigation, including genetic profiling and real-time assessment of the dynamic changes of biomarker expression and mutations during therapy. While the gut microbiome has been clearly shown to impact immune responsiveness, lack of consistent data with similar species across cancer types emphasizes the need for further research to better understand this complex relationship. Additionally, challenges persist in identifying reliable prognostic indicators of response to ICI and the role of immunomodulation and immune profiling in guiding the selection of treatment regimens. Correlation with mechanisms of resistance observed in more novel cellular-based therapies may also provide insight into better understanding the mechanisms we describe here. Although immunotherapy is generally well tolerated, novel ICIs are currently being developed to enhance therapeutic benefits for patients who have shown resistance to current immunotherapy regimens, addressing unmet needs in the treatment landscape.

## Data Availability

The data that support the findings of this study are available on request from the corresponding author, MAB. The data are not publicly available due to production of illustrations by an author and protection of independent work.
